# Perinatal Occurrence and Epidemiological Significance of *Staphylococcus aureus* in Local Sheep Breeds

**DOI:** 10.3390/ani16030400

**Published:** 2026-01-27

**Authors:** Agata Hahaj-Siembida, Aneta Nowakiewicz, Mariola Bochniarz, Aleksandra Trościańczyk, Marcelina Osińska, Anna Tracz, Andrzej Junkuszew, Karina Savvulidi Vargova, Monika Greguła-Kania

**Affiliations:** 1 Sub-Department of Veterinary Microbiology, Department of Preclinical Veterinary Sciences, University of Life Sciences, 20-950 Lublin, Polandaneta.nowakiewicz@up.edu.pl (A.N.); mariola.bochniarz@up.edu.pl (M.B.); aleksandra.troscianczyk@up.edu.pl (A.T.); anna.tracz@up.edu.pl (A.T.); 2 Department of Animal Breeding and Agricultural Advisory, Faculty of Animal Sciences and Bioeconomy, University of Life Sciences, 20-950 Lublin, Poland; andrzej.junkuszew@up.edu.pl; 3 Institute of Pathological Physiology, First Faculty of Medicine, Charles University, 128 53 Prague, Czech Republic; vargova.mmbd@gmail.com

**Keywords:** *Staphylococcus aureus*, drug resistance, virulence genes, sheep, MLST, ADSRRS-fingerprinting

## Abstract

*Staphylococcus* are common bacteria that can be a component of the natural microbiota of the body of human and animal hosts or cause serious infections. The aim of this study was to analyze the presence of *S. aureus* in samples from two sheep breeds (Świniarka and Uhruska), assess the antimicrobial susceptibility and virulence profile of these microorganisms, and perform molecular comparative analysis of these bacteria. One hundred and three isolates of *S. aureus* strains were obtained (including five MRSA strains) and the highest phenotypic resistance to tetracycline (46.6%), clindamycin (45.6%), erythromycin (39.8%), and penicillin (31.1%) was confirmed. The *tetL* gene (27.2%) and the *seB* gene (44.7%) were the most frequently detected in strains. Three new sequence types in the MRSA isolates (ST 9313, ST 9314, ST 9315) were identified. In conclusion, our results confirm that sheep are significant reservoirs of *S. aureus*, especially MRSA strains, varying in terms of both resistance and virulence and with the potential to spread between individuals in the same herd, which may pose a potential health problem.

## Simple Summary

Sheep are farm animals that provide numerous goods such as wool, meat, and milk, and animal-derived foods constitute an important part of the human diet. *Staphylococcus aureus* can spread between farm animals and humans, posing a public health threat and increasing the already high level of multi-drug resistant bacterial strains in both hospitals and the environment. This research shows that healthy sheep are a reservoir of *S. aureus* strains including methicillin-resistant strains (MRSA) and multi-drug resistance (MDR). The oral cavity should be considered the most representative site for sample collection. The level of *S. aureus* colonization is not constant and varies with the breed and physiological condition. During pregnancy, the level of *S. aureus* colonization significantly increases. In total, 46.6% of all tested strains met the criteria for multi-drug resistance. The primitive breed, Świniarka, is more resistant to both colonization and infection, which in turn is associated with less frequent antibiotic use in these breeds and lower levels of resistance in the colonizing strains. The presence of MRSA strains was detected at low levels in both breeds.

## 1. Introduction

Sheep were domesticated as one of the first farm animals in southwest Asia around 10,000 to 8000 BC. Originally, sheep were domesticated from the Asian mouflon (*Ovis gmelini*) whose occurrence range extended from Turkey to eastern Iran. From the very beginning, humans obtained wool, pelts, meat, and milk from sheep herding, but these small animals also played an important role in society, especially in Asia and later in Europe. Finally, domestic sheep (*Ovis aries*) were spread around the world by humans. The sheep population is 1.2 billion worldwide [1], and there are currently hundreds of breeds of domestic sheep worldwide as well as many mouflon lineages (such as wild relatives of domesticated animals, including wild sheep) and five other species of wild sheep [2,3,4,5,6]. Sheep breeding also has a long tradition in Poland. Unfortunately, sheep currently constitute only 1% of the number of animals bred in Poland and are used mainly for the production of meat and milk [7,8]. There are over a dozen sheep breeds in specific or mixed types of use, with 17 native breeds covered by the Genetic Resources Protection Program, and these breeds include, among others, the Świniarka breed and the Uhruska breed (Polish Lowland Sheep) [7,8,9]. The Świniarka breed, i.e., one of the oldest breeds of sheep, is resistant to numerous diseases and well adapted to various environmental conditions. In comparison with the Świniarka breed, the Uhruska breed is a refined breed with body weight of 55–60 kg, high-quality wool, and high fertility. It is a native variety bred in the central-eastern region of Poland. Sheep of this breed make good use of agricultural feed and natural pastures [7,8,9,10].

The specific status of *S. aureus* as a commensal isolated also from clinically healthy animals makes this bacteria a frequent source of antibiotic resistance. *S. aureus*, especially methicillin-resistant strains (MRSA), can spread between farm animals and humans, posing a public health threat and increasing the already high level of multi-drug resistant bacterial strains in both hospitals and the environment [11,12,13,14]. In 2024, the World Health Organization published and updated the Bacterial Priority Pathogens List [15], and MRSA strains are still included in the high-priority pathogen category. Moreover, the Global Burden of Disease study reported that approximately 50% of fatal infections in highly developed countries are caused by multi-drug resistant *S. aureus* and *E. coli* [15]. The WHO warns that, in the near future, there may be an increase in the number of cases of infections with strains for which medicine will no longer have any effective therapeutic option. Bacterial infections, especially those caused by *S. aureus*, pose a huge problem associated with its causative role in disease in animals or the high level of drug resistance and a risk for the food industry. Products, such as milk or meat, containing bacteria can pose a serious threat to public health. With its relatively high resistance to unfavorable environmental conditions, *S. aureus* is able to survive outside the host for a long time; therefore, it is one of the most common bacteria contaminating food of animal origin, including sheep [16,17,18].

Therefore, a comprehensive approach combining global surveillance including farm animals, which can be frequent carriers of *S. aureus*, is needed.

To address the contemporary scientific challenges, such as the presence of *S. aureus* in the environment, understand the basics of resistance mechanisms, and ultimately develop effective therapies to combat the problem of microbial resistance, the aim of this study was to compare the presence of *S. aureus* in different biological samples from two sheep breeds (Świniarka and Uhruska) collected from animals in different physiological states and to analyze the level of drug resistance and virulence of these microorganisms.

## 2. Materials and Methods

### 2.1. Description of Animals and Collection of Materials

The Local Ethics Committee for Animal Experimentation in Lublin, Poland approved this study (License No 95/2023). The research material came from ewes of two breeds that are native in Poland: Uhruska and Świniarka. The Świniarka breed is generally considered a primitive type, and the Uhruska breed exhibits high fertility and meat performance. The animals were kept in identical nutritional and environmental conditions described previously [8] in an indoor-pasture system on an experimental farm belonging to the University of Life Sciences in Lublin (51°11′60.00″ N 23°15′60.00″ E), located in south-eastern Poland Bezek. Mating lasted six weeks, starting in September for Uhruska breed ewes and in October for the Świniarka breed, and lambing lasted from mid-January to February for the Uhruska ewes and from February to March for the Świniarka breed. The ewes were the same age (2–3 years old). The inclusion of only healthy sheep in the experiment was based on the results of a general clinical examination performed by a veterinarian. The research material from the ewes was collected as swabs and milk.

Swabs were collected from four anatomical sites (skin, external ear canal, mucus membranes of anus, and mouth) in ewes, and exclusively from the oral cavity in lambs. Oral swabs were obtained from the hard palate and tongue, while skin swabs were collected from the right shoulder blade after parting the wool. Samples taken from all sites were collected using sterile swabs. Each swab was rubbed five times against the mucosal surfaces or skin with firm pressure. Samples from adult ewes were collected individually during four separate sampling periods. In each sampling period, 50 adult sheep were examined (25 individuals from each breed). The material was collected by a veterinarian during a routine examination in the following periods: I—before pregnancy, II—at the beginning of pregnancy, III—at the end of pregnancy (two weeks before delivery), IV—after giving birth, during lactation (one week after delivery). Swabs from one-week-old lambs (50 in total) were collected only once (after parturition). Milk samples were collected from ewes after parturition (25 samples from each breed). A total of 900 microbiological samples were collected, including 850 swabs (800 from adults and 50 from lambs) and 50 samples of milk. Milk samples of 5 mL volume were collected according to a procedure described previously [8].

### 2.2. Isolation and Identification of Staphylococcus aureus

The isolation of *S. aureus* from the swabs and the milk samples was performed according to the procedure described by Hahaj-Siembida et al. [8]. From each positive sample, one morphologically typical colony was selected, subcultured on nutrient agar, and subjected to further biochemical identification [8]. All isolates identified as *Staphylococcus* spp. based on morphological and biochemical characteristics were subsequently confirmed using molecular methods, including analysis of *nuc* gene polymorphism [8,19]. To ensure independence of observations, only one isolate per animal was included in the statistical analysis, regardless of the sampling site or period.

### 2.3. Disk Diffusion Test (Kirby–Bauer Test)

The drug resistance of the *S. aureus* strains was assessed with the disk diffusion method [20]. Thirteen antimicrobial substances were selected in the following concentrations: erythromycin 15 μg, cefoxitin 30 μg, gentamicin 10 μg, rifampicin 5 μg, quinupristin/dalfopristin 15 μg, clindamycin 2 μg (used alone and in tests detecting induced clindamycin resistance in *Staphylococcus* spp.), penicillin G 10 U, sulfamethoxazole/trimethoprim 25 μg, tetracycline 30 μg, nitrofurantoin 300 μg, linezolid 30 μg, ciprofloxacin 5 μg, and chloramphenicol 30 μg (Thermo Scientific™ Oxoid™, Waltham, MA, USA). The results were evaluated in accordance with the recommendations of the Clinical and Laboratory Standards Institute [21,22]. The reference strain *S. aureus* ATCC 25923 was used as a quality control. The multi-drug resistance of the strains was classified based on the criteria described by Magiorakos et al. [23]

### 2.4. Detection of Resistance and Virulence Genes

Bacterial DNA isolation was performed using the commercial Gram Plus and Yeast Genomic DNA Purification Kit (Eurx, Gdansk, Poland) according to the manufacturer’s instructions.

The following resistance genes were selected: *mec*A, *mec*C, *bla*Z (beta-lactam resistance), *erm*A, *erm*B, *erm*C, *msr*A (macrolide resistance), *cat* (*pC223*), *cat* (*pC221*), *cat* (*pC194*) (phenicol resistance), *tet*K, *tet*M, *tet*L (tetracycline resistance), *acc*(*6′*)-*le*, and *aph*(*3*)-*IIIa* (aminoglycoside resistance). The primer sequences and reaction conditions were identical to those described previously [8,24,25,26,27,28,29].

The following virulence genes were selected: *se*A, *se*B, *se*C, *se*D, and *se*E encoding enterotoxins (SEA-E), *tst* encoding toxic shock syndrome toxin-1 (TSST-1), *PVL* encoding leukocidin (components S and F), and *LukE-LukD* encoding leukotoxin LUKE/LUKD [8,29,30].

### 2.5. Determination of the Minimal Inhibitory Concentration (MIC) of Methicillin-Resistant S. aureus Strains

The susceptibility of MRSA strains confirmed by the presence of the *mec*A and/or *mec*C genes was assessed using the microdilution method according to the Clinical and Laboratory Standards Institute guidelines [21,22]. The minimum inhibitory concentrations in the range of 0.25–128 µg/mL were analyzed for eleven selected antimicrobials: erythromycin, oxacillin, gentamicin, rifampicin, clindamycin, penicillin, tetracycline, nitrofurantoin, ciprofloxacin, chloramphenicol, and vancomycin (Sigma-Aldrich, Darmstadt, Germany). The reference strain *S. aureus* ATCC29213 was used as a positive control.

### 2.6. Multi Locus Sequence Typing (MLST) of MRSA Strains

The MLST analysis was performed for seven housekeeping genes: *arcC*, *aroE*, *glp*, *gmk*, *pta*, *tpi*, and *yqiL*. Specific multiplex-PCR reactions were performed according to the protocol described elsewhere (PubMLST https://pubmlst.org/organisms/staphylococcus-aureus/primers, accessed on 21 January 2026). The reactions were performed in a volume of 25 µL using Silver Taq MIX (Syngen Biotech, Wrocław, Poland) in a T100 thermocycler (Bio-Rad, Hercules, CA, USA). PCR products were purified using the Clean-up kit (A&A Biotechnology, Gdańsk, Poland). DNA sequencing was performed using the Sanger method by Genomed S.A. (Warsaw, Poland). The obtained nucleotide sequences were compared with alleles in the PubMLST database (https://pubmlst.org/, accessed on 21 January 2026). Subsequently, based on allele combinations, STs and clonal complexes were assigned. Nucleotide sequences of the genes were deposited in GenBank (https://www.ncbi.nlm.nih.gov/genbank/, accessed on 21 January 2026).

### 2.7. ADSRRS-Fingerprinting of S. aureus Strains

*S. aureus* strains isolated from the two sheep breeds were genotyped using the ADSRRS-fingerprinting (amplification of DNA fragments surrounding rare restriction sites) method according to the protocol described by Krawczyk et al. [31] with slight modifications [32,33]. Initially, genomic DNA was digested with two restriction enzymes: XbaI (10U) and BglII (5U) (Thermo Scientific, Waltham, MA, USA) at 37 °C for 1 h. Then, ligation reactions were carried out using T4 DNA ligase at 25 °C for 1 h (Thermo Scientific, Waltham, MA, USA) and appropriate adapters were used. Subsequently, after heat inactivation at 70 °C for 5 min, PCR reactions were performed in a volume of 25 µL containing 1 µL of ligation mix, Silver Taq MIX (Syngen Biotech, Wrocław, Poland), and 50 pmol of each primer (Genomed S.A., Warsaw, Poland). The PCR reactions were performed in a T100 thermal cycler (Bio-Rad, Hercules, CA, USA) using a personalized thermal profile (94 °C—5 min, 72 °C—5 min, 94 °C—5 min, 22 cycles (94 °C—30 s, 60 °C—30 s and 72 °C—90 s), and 72 °C—7 min). Subsequently, PCR products were visualized in an 8% polyacrylamide gel and stained with ethidium bromide. The electrophoretic profiles were documented in the GelDoc Go System (Bio-Rad, Hercules, CA, USA). Subsequently, the analysis of similarity of electrophoretic profiles was performed with the UPGMA method using the BIO-1D++ 11.9 gel analysis software (Vilber, Lourmat, Collégien, France) based on the Jaccard correlation coefficient (tolerance and optimization 1%) [32,33].

### 2.8. Statistical Analysis

The statistical analysis was performed using Statistica software version 13.1. Due to the categorical nature of the data and limited group sizes, Fisher’s exact test (*p* < 0.05) was used to assess statistically significant differences between strains isolated from the two breeds (Uhruska and Świniarka), including phenotypic antimicrobial resistance and the presence of resistance and virulence genes.

## 3. Results

### 3.1. Isolation and Identification of S. aureus

In total, 103 strains of *S. aureus* were isolated from the two sheep breeds, including 66 isolates from the Uhruska breed and 37 isolates from the Świniarka breed. All strains were confirmed as *S. aureus* by the analysis of the *nuc* gene polymorphism. The largest number of strains, regardless of the breed, was isolated from the mouth and constituted 59.2% of all *S. aureus* isolated. The other *S. aureus* strains were isolated from the rectum (14.6%) and the skin (9.7%), and the fewest isolates, constituting 6.8%, were obtained from the external ear canal. In addition, only 9.7% of the *S. aureus* isolates originated from sheep’s milk (Table 1).

Depending on the period of collection, the number of isolates in which *S. aureus* was detected varied (Table 2). Before pregnancy, *S. aureus* was detected only from the Uhruska sheep, whereas no *S. aureus* was detected in any of the Świniarka sheeps at that time. However, at the beginning of pregnancy, 29 isolates were detected in both breeds, with 14 isolates from the Uhruska breed and 15 isolates from the Świniarka sheep. In the samples collected at the end of pregnancy, 35 isolates of *S. aureus* were detected, with 18 isolates of *S. aureus* from the Uhruska breed and 17 isolates from the Świniarka breed. In contrast, after parturition/during lactation, in total, 16 S. aureus isolates from the ewes were detected (12 isolates from the Uhruska sheep and four isolates from the Świniarka breed). Moreover, during this period (after parturition/during lactation), six isolates (Uhruska breed) were detected from lambs and 10 isolates were detected in the milk of ewes (nine strains of Uhruska breed and one strain from Świniarka sheep). In general, our results showed that, from 32 animals (including 19 Uhruska sheep and 13 Świniarka ewes), we isolated more than one S. aureus strain (Supplementary Table S1).

### 3.2. Phenotypic Drug Resistance of S. aureus

All 103 *S. aureus* strains isolated from the ewes, lambs, and milk were tested. We showed resistance to all antimicrobials in strains isolated from the Uhruska sheep and to eight of the thirteen antimicrobials tested in strains isolated from Świniarka (Table 3). The highest percentage of phenotypic resistance among strains isolated from both breeds was recorded in the case of tetracycline (46.6%), clindamycin (45.6%), erythromycin (39.8%), penicillin (31.1%), and gentamicin (24.3%). Our study also showed resistance to oxacillin in five isolates: two originating from an ewe and a lamb of the Uhruska breed and three from the Świniarka sheep. All MRSA isolates originated from different animals. In addition, each isolate exhibited phenotypic resistance to the other antimicrobials tested.

The statistical analysis revealed significant differences in the phenotypic resistance to chloramphenicol (resistance to this antibiotic was demonstrated only in strains isolated from the Uhruska breed). The isolates from the same breed also showed a significantly higher percentage of strains resistant to gentamicin. However, the opposite trend was observed for erythromycin resistance, which was significantly higher in strains isolated from the Świniarka breed (Table 3).

Multi-drug resistance was demonstrated in 48 strains (46.6% of all tested strains), 70.8% of which were isolated from the Uhruska breed. In the MDR group, resistance to three antimicrobials was most frequently observed (*n* = 24, 50%), 12 strains were resistant to four antimicrobials (25%), while eight and four strains (16.6% and 8.3%) were resistant to five and six antimicrobials, respectively. Non-susceptibility to any of the tested antimicrobials was noted in 23 of all tested strains (22.3%), while resistance to only one or two antimicrobials was observed in 32 (31.1%) strains which were defined as non-MDR.

### 3.3. Occurrence of Resistance and Virulence Genes in S. aureus

The dominant gene determining resistance to tetracycline was *tet*L, which was present in 27.2% (*n* = 28) of the strains. The *tet*K gene was frequently associated with resistance in the tetracycline-resistant strains (20.4%, *n* = 21). Phenotypic resistance to penicillin was accompanied by the *bla*Z gene in 11.7% (*n* = 12). The other genes tested were detected in single strains (Table 4). In five of the tested strains, *mec*C genes were detected, which were accompanied by the presence of *mec*A genes in two cases. Their presence was accompanied by phenotypic resistance only in the case of two strains. Interestingly, the *mecA*, *tetM*, *msrA*, and *ermB* genes were detected exclusively among strains isolated from the Uhruska breed. However, statistically significant differences in the level of resistance genes in strains isolated from both breeds were noted for *tet*K end *erm*B, which were more often present in strains isolated from the Świniarka breed. The *S. aureus* strains isolated from the Uhruska breed and the Świniarka sheep were also examined for the presence of virulence genes. The presence of three genes was dominant: two genes, *se*B and *se*C, encoding enterotoxins (44.7%, *n* = 46 and 32%, *n* = 33, respectively) and genes encoding leukotoxin *LukE-LukD* (30%, *n* = 31). However, only *S. aureus* strains isolated from the Uhruska breed possessed the *se*A gene and the *pvL* gene. On the other hand, only single strains of *S. aureus* isolated from the Świniarka breed had the *se*D, *se*E, and *tst* genes (Table 5). However, there were no statistically significant differences between the presence of virulence genes in strains isolated from the Uhruska and Świniarka breeds.

### 3.4. Characterization of MRSA Strains

#### 3.4.1. MIC Values of Methicillin-Resistant *Staphylococcus aureus* Strains

The criterion for qualifying for the evaluation was the presence of the *mec*C and *mec*A genes in the strains tested. In total, five MRSA strains were characterized, including two isolated from the Świniarka breed and three strains from the Uhruska breed (Table 4). The susceptibility profiles of the methicillin-resistant *S. aureus* strains were additionally assessed using the microdilution method. All MRSA strains showed a penicillin resistance profile with MIC values of 64 μg/mL (two strains) and 128 μg/mL (three strains). Three MRSA strains showed a vancomycin resistance profile with MIC values of 32 μg/mL (two strains) and 64 μg/mL (one strain). In addition, two MRSA strains showed a tetracycline resistance profile with an MIC value of 128 μg/mL. Similarly, two MRSA strains also showed resistance to chloramphenicol with an MIC value of 32 μg/mL. Only two strains showed phenotypic resistance to oxacillin with an MIC value of 8 μg/mL despite the presence of the *mecA* or *mecC* gene. Three of the *S. aureus* strains tested were phenotypically susceptible to oxacillin with an MIC of 2 μg/mL. In addition, one isolate showed resistance to erythromycin and one was resistant to gentamicin with MIC values of 8 μg/mL and 16 μg/mL, respectively (Table 6). All the MRSA strains tested were susceptible to ciprofloxacin, rifampicin, and nitrofurantoin.

#### 3.4.2. MLST of Methicillin-Resistant *S. aureus* Strains

The multilocus sequence typing analysis was performed on methicillin-resistant *S. aureus* (Table 6). Based on the MLST analysis, the MRSA isolates were classified into five different STs (sequence types), including three new ones, which had not been reported before. The new STs identified in the *S. aureus* isolates were ST 9313, ST 9314, and ST 9315. Two strains had STs already known in the database, i.e., ST 1660 (*n* = 1) and ST 8420 (*n* = 1). Interestingly, strains with two different STs (9314 and 8420) originated from the same individual (Świniarka breed), were isolated from the oral cavity at the same time (period IV), and had an identical profile to the tested genes and a very similar phenotypic resistance profile (the difference was only at the intermediate level to erythromycin). Moreover, their differentiation was also confirmed by their affiliation to other ADSRRS profiles (Table 7). The nucleotide sequences were deposited in GenBank under the following accession numbers: *tpi*: *PQ130080.1–PQ130076.1*, *aroE*: *PQ119410.1–PQ119406.1*, *arcC*: *PQ119398.1–PQ119394.1*, *glpF*: *PQ119422.1–PQ119418.1*, *gmk*: *PQ130056.1–PQ130052.1*, *pta*: *PQ130068.1–PQ130064.1*, *and ygiL*: *PQ130092.1–PQ130088.1.*

### 3.5. ADSRRS-Fingerprinting Results and Similarity of S. aureus Isolates

The ADSRRS-fingerprinting covered all the 103 isolated strains. Based on the obtained ADSRRS-fingerprinting results, a dendrogram of similarity was generated (Figure S1), where thirty-two different profiles overlapping 90% were distinguished. The analysis showed that 14 profiles grouped three or more strains, and profiles D, H, J, P, X, and c grouped strains exclusively isolated from the Uhruska breed (Table 7). In the case of profiles grouping strains in pairs, it was shown that profiles B, M, and N grouped only strains from the Świniarka breed, while profiles T and X grouped only strains from the Uhruska breed. Seven and six profiles were specific only to single strains obtained from the Uhruska breed and the Świniarka sheep, respectively (Table 7). Among the strains isolated from the Uhruska breed, in four cases, similarity exceeding 90% was demonstrated between two or even three strains isolated from the same animals from different body sites or from the same sites but isolated at different times (strains from sheep II, V, VI, and XX). Moreover, in the case of lambs from the same mother (Uhruska breed, IV), two strains were assigned to the same Y genomic profile but had different profiles of both resistance and virulence. In the case of strains isolated from the Świniarka breed, a similar situation was also observed in three groups of strains (strains from sheep IX, XV, and XXIV): in two cases, highly similar strains were isolated from the skin and mouth or from the skin and external ear canal. In the third case, the strains were isolated at the same time from the mouth of the same animal, but despite the high similarity exceeding 90% and the identical profile of detected genes, they differed in their phenotypic resistance profile. In the case of some strains isolated from the same animals (Świniarka breed I and IV), their profiles (M and N) were also similar to each other, although at a slightly lower level than 90% (87%). We observed a similar situation for two other strains from animal IV (Uhruska breed), which were classified into two adjacent profiles (T and U), and the level of similarity was 85%. As expected, the MRSA strains showed no similarity among each other; the strains were classified into different profiles: J, R, Y, b, and d.

An interesting phenomenon was the demonstration of typical “milk strains” profiles, including strains isolated exclusively from the milk of different ewes belonging to the same breed (profile J and c) and even from animals belonging to both breeds (profile b, including two strains isolated from the Uhruska breed and two strains isolated from the Świniarka breed). Furthermore, 16 isolates showed differences in features related to resistance genes, virulence genes, or phenotypic resistance to a given antibacterial substance, compared to samples from the same site and sampling date. However, four isolates showed differences only in the virulence genes. Moreover, in one case of a Świniarka sheep, samples collected on the same date (date III—at the end of pregnancy) but from different sites (ear, anus, mouth) showed that the tested isolates possessed the same resistance and virulence genes. However, in this individual sheep, all strains were phenotypically resistant to tetracycline, with two of the three being resistant to linezolid and only one being resistant to cefoxitin. Furthermore, some strains showed significant similarity to other strains from the same location or date, and their similarity consisted of the presence of the same resistance or virulence genes or phenotypic resistance profile. However, the vast majority of strains from a single individual showed significant differences in their pool of the tested resistance and/or virulence genes or resistance phenotypic profiles, compared to isolates from the same site but different locations or even from the same site.

## 4. Discussion

At present, the total number of all bacterial species, both pathogens and commensals, in all habitats worldwide may exceed one million [34]. Many of these species are zoonotic microorganisms occurring both in animals and in their habitats. The most numerous genera in this group include *Staphylococcus* spp.; *Salmonella* spp.; *Campylobacter* spp.; *Listeria* spp.; *Enterococcus* spp., and *Escherichia coli* [34,35,36]. Some of these species may also be a potential threat to public health, including the genus *Staphylococcus*, with particular emphasis on *S. aureus*. As is known, small ruminants such as sheep are a reservoir of this species, which was also confirmed by our previous studies [8]. The specific dual status of this species (as a commensal or a pathogen responsible for a wide range of infections and foodborne diseases) is a frequent target of research as a cause of disease but a less frequent target of monitoring studies in both humans and various animal species. In the case of sheep and goats, scientific reports typically focus on the analysis of this microorganism as a pathogen responsible for mastitis, respiratory infections, or septicemia in lambs [37,38,39]. However, monitoring studies examining the occurrence and characteristics of *S. aureus* strains isolated from clinically healthy animals are conducted much less frequently in these animal species. Our research confirmed that, regardless of the breed, *S. aureus* is commonly present in healthy sheep, although at very varying levels depending on the breed and the site of sample collection. Saad et al. [40] showed a level of 80% of carriage of *S. aureus* isolates from three different body regions in Egyptian goat and sheep, while Vautor et al. [41] confirmed *S. aureus* carriage in nostrils in 29% of healthy sheep. Agabou et al. [42], in turn, showed a 44% *S. aureus* carriage rate also in material collected from the same site. Therefore, in our research, we decided to employ an innovative strategy for collecting material; while isolating the strains, we took into account as many as four different sites and four different periods related to the physiological pregnancy cycle in two sheep breeds, including one that is almost native and therefore theoretically more resistant to the colonization by microorganisms. The oral mucosa turned out to be colonized by *S. aureus* the most frequently, with nearly 60% of all *S. aureus* strains obtained from this site and two-thirds of these strains originating from the native Uhruska breed. Similarly, in the case of the milk isolates, nine out of ten strains originated from the milk of the Uhruska sheep. In the case of the other isolation sites, the percentage remained relatively low, ranging from 14.6% from the rectum to only 6.8% from the external ear canal, and the level of isolation was comparable for both breeds. The obtained results indicate a relatively high rate of *S. aureus* colonization of the oral mucosa of clinically healthy sheep and suggest that the oral cavity should primarily be considered when selecting the most representative site for sample collection. When collecting samples from the anus, skin, or external auditory canal, the colonization results may be significantly underestimated. Our study also showed a twice higher rate of *S. aureus* colonization in the Uhruska breed, compared to the Świniarka sheep, which may indicate a significantly higher level of defense mechanisms related to colonization in clinically healthy animals of the native breed.

Available studies indicate that the most common sites for sample collection in sheep are the udder or teat skin and the nasal cavity or rectum, as opposed to the oral cavity [43], which, as shown in our studies, is the main site of colonization by *S. aureus*. The presence of *S. aureus* in the oral cavity likely favors vertical transmission of this microorganism between the mother and the newborn lamb, as licking is part of natural behavior associated with establishing a healthy mother–lamb bond, odor learning, and encouraging colostrum intake. Licking is also essential for hygiene and offspring recognition within the flock. A study conducted by Campos et al. [44] on clinically healthy volunteers showed that, similarly to sheep, the human oral cavity is a very common site of colonization by *S. aureus* strains. The nasal and oral cavities can be colonized independently by different strains belonging to the *S. aureus* species, for example, with different resistance profiles, which is extremely important from an epidemiological point of view. Our studies also revealed the presence of diverse *S. aureus* strains occurring in the same individual but isolated from different sites, confirming that the isolation of only one potentially representative strain does not reflect the full level of resistance and/or virulence of *S. aureus* strains occurring in a given host. Our research also confirmed that the level of *S. aureus* colonization in sheep is not a constant value and varies not only depending on the breed but also on the physiological state associated with pregnancy. We demonstrated that, during pregnancy, regardless of its stage (initial or final), the level of *S. aureus* colonization significantly increases threefold compared to the pre-pregnancy period, and decreases twofold after parturition, compared to the last period of parturition, which was particularly evident in the Świniarka breed. This may indicate reduced involvement of the mother’s defense mechanisms during physiological pregnancy, which translates into a higher percentage of colonization with commensal microorganisms such as *S. aureus* or a change in their pathogenic status and ability to cause infection. It is also worth noting that the absence of *S. aureus* in the Świniarka breed before pregnancy may be due to the fact that this primitive breed is more resistant to colonization, which has already been shown in our previous studies [8].

The *Staphylococcus* genus is isolated from 70% of clinical mastitis cases, with *S. aureus* being the most frequently reported species [39]. The combination of high levels of antibiotic resistance and a rich set of virulence factors makes *S. aureus* isolated from sheep and their milk a threat to public health. Our study demonstrated a relatively high level of drug resistance among the isolated strains, with the highest levels of resistance to clindamycin, erythromycin, tetracycline, and penicillin, which was consistent with the resistance profile reported by other authors [45,46]. Furthermore, 46.6% of all the strains tested met the criteria for multi-drug resistance. This phenomenon may be related to the frequent use of penicillins, tetracyclines, and aminoglycosides in sheep flocks during the treatment of mastitis, pneumonia, or diarrhea in lambs, which favors the development of selection pressure [47]. In the case of penicillin resistance, only half of the penicillin-resistant strains were found to possess the *bla*Z gene responsible for penicillinase production. However, it has been shown that there are currently a number of other mechanisms associated with resistance to beta-lactams, in addition to those detected in this study, i.e., the presence of the *bla*Z, *mec*A, and *mec*C genes [48]. Therefore, the correlation between the occurrence of the most common genes and phenotypic resistance may be low.

We observed a similar phenomenon in strains with phenotypic resistance to oxacillin and *mec* genes. Of the five oxacillin-resistant strains, the presence of the *mec*A and/or *mec*C genes was demonstrated in only two. However, in the case of the three other strains exhibiting phenotypic resistance only to penicillin, we demonstrated the presence of the *mec* gene(s), classifying the strains as MRSA, and their prevalence in the studied group of animals was 4.9%. It should be noted that the prevalence of MRSA strains is quite variable; they are usually most frequently reported in mastitis cases [49], even at levels exceeding 40%. On the other hand, they may not be present at all in clinical infections [50]. Interestingly, although *mec*A is the dominant gene determining MRSA, studies increasingly show the presence of both genes in the same strain, even among isolates of human origin [51]. In our study, the presence of both genes did not demonstrate a cumulative effect since one strain did not exhibit phenotypic resistance to oxacillin and the other had an MIC of 8 µg/mL; therefore, the epidemiological significance of this phenomenon has not yet been determined. In the case of MRSA strains carrying the *mec* gene(s), based on the MIC values, we also demonstrated resistance to vancomycin, the level of which classified the tested strains as VISA (vancomycin-intermediate *S. aureus* MIC 4–8 µg/mL) or VRSA (vancomycin-resistant *S. aureus*, MIC > 8 µg/mL). The presence of strains resistant to glycopeptides poses a significant therapeutic challenge. VRSA strains have already been reported in farm animals, mainly from cases of bovine mastitis, even at levels exceeding 10%, which indicates the progressive spread of resistance to this group of drugs among *S. aureus*, despite the discontinuation of the use of avoparcin as a growth promoter in animals [52,53]. The high percentage of gentamicin-resistant strains exceeding 20% may in turn be a result of the use of kanamycin in animals, which may induce cross-resistance to other aminoglycosides except for streptomycin. The presence of the acc(6′)-Ie- gene, encoding a bifunctional enzyme that inactivates gentamicin, kanamycin, tobramycin, netilmicin, and amikacin by phosphorylation and/or acetylation [48], was confirmed in seven of the 25 gentamicin-resistant isolates. High-level phenotypic resistance to erythromycin and clindamycin is very common, as in our study, and is usually associated with the presence of enzymes that confer inducible or constitutive resistance to MLSB antibiotics [54]. However, in our study, only six strains demonstrated the presence of the *erm*B gene, indicating constitutive resistance to MLSB antibiotics, and three strains exhibited a different mechanism: the presence of the *msr*A gene encoding the macrolide efflux mechanism, common in *Staphylococcus* strains [55]. Our studies also confirmed that the plasmid-borne *tet*K gene is the most prevalent tetracycline resistance gene among *Staphylococcus* and encodes a similar resistance mechanism to *tet*L, the second most common gene [56]. Due to its mobility, it spreads quite easily among strains even without selective pressure, determining high levels of tetracycline resistance, as also demonstrated in our studies. Only three of the seven chloramphenicol-resistant strains showed the presence of the *cat* gene encoding acetyltransferase. Despite the ban on the use of non-fluorinated phenicols in animals, genes encoding acetyltransferase are quite common among *Staphylococcus* and *Enterococcus* strains in farmed and wild animals due to their plasmid-borne nature and ease of spread [32].

The virulence gene profile was quite similar to *S. aureus* strains isolated from sheep, especially from mastitis cases [37]. In most cases, the dominant gene encoding enterotoxins is *se*C, often occurring in combination with *se*B or other genes, depending on the geographic region [50,57,58,59]. The gene encoding the leukotoxin *LukE-LukD* was also present in over 30 strains tested. The presence of enterotoxin-positive strains, especially in sheep milk due to the high thermoresistance of enterotoxins, poses a public health threat [37]. Furthermore, all proteins encoded by these genes are involved in the immune response, acting as superantigens and overactivating T lymphocytes, leading to massive cytokine release. LukED, a protein toxin that forms pores in the membranes of immune cells (neutrophils, monocytes), plays a role in evading the host immune response. In the case of *S. aureus* strains isolated from small ruminants, as in our studies, only single strains possessing the gene encoding Panton Valentin toxin and toxic shock syndrome toxin are usually detected [60]. The TSST toxin, like enterotoxins, has cytokine storm properties, which underlie the process of triggering toxic shock syndrome [61]. *S. aureus* producers of Panton Valentine toxin (leukocidin) are usually associated with soft tissue and also life-threatening infections in humans. However, in the case of leukocidin, species specificity is observed (the effect of killing neutrophils of a specific host species) [62]. On the other hand, cases of animals involved in the transmission of PVL-positive strains in humans have been confirmed [63,64]. Furthermore, in hospital-acquired infections, these toxins are often associated with MRSA strains, mainly community-associated (CA-MRSA) [65]; therefore, in the case of zoonotic strains, the epidemiological significance of these toxins should be further investigated.

The MLST analysis used in our study for MRSA typing allowed the identification of three new sequence types in the MRSA isolates (ST 9313, ST 9314, ST 9315), which, on the one hand, indicates continuous evolution within the *S. aureus* species. On the other hand, known sequence types were also detected, such as ST 1660 (*n* = 1), which was so far detected only in equine *S. aureus* isolates from Denmark [66], Spain [67], and Germany [68,69], and ST 8420 (*n* = 1), which was uniquely detected in milk isolates in New Zealand, as reported on the MLST website (https://pubmlst.org/multilocus-sequence-typing, accessed on 21 January 2026). ST 1660 was isolated from both MRSA and MSSA strains, but horses were the source of these strains. Interestingly, the animals studied in this study had no contact with this animal species, confirming that the typically “equine” sequence type can also adapt to other animal species or humans. It may have been merely transient colonization, as the strain was isolated from a young animal, a lamb with non-established microbiota [70].

Our studies also demonstrated a significant diversity of strains based on the method of total genomic DNA analysis (ADSRRS-fingerprinting). We primarily demonstrated that the same animal without clinical symptoms can be colonized by several genetically diverse *S. aureus* strains (e.g., animal IV from the Uhruska breed was colonized by strains belonging to the K, U, Y, d, T, X, and f profiles) and that different types can colonize not only different sites, such as the oral cavity, skin, or rectum, but even the same site, e.g., the oral cavity. We confirmed this phenomenon not only through the different ADSRRS profiles but also through the different phenotypic and genotypic resistance and virulence profiles of strains from the same individual. Our chosen research strategy seems appropriate. In the case of resistance, as our studies also demonstrated, genes present in the genome are not always expressed. This was particularly evident in MRSA strains, in which, despite phenotypic susceptibility, we demonstrated the presence of the mec gene(s) [71]. Combined with MLST analysis, this research strategy offers the opportunity to identify potentially new emergency clones that could initially spread between animals and then, if they adapt to new hosts (as is typical for S. aureus), pose a further threat to public health [72,73].

Furthermore, our studies also demonstrated the presence of strains with a level of similarity of at least 90%, indicating extensive cross-colonization by *S. aureus* strains among different animals but belonging to the same herd. On the other hand, only *S. aureus* isolates from the Świniarka breed or only *S. aureus* isolates from the Uhruska breed showed the same profile, which confirms the spread of *S. aureus* strains among different individuals but only of the same sheep breed. However, this does not rule out the transmission of strains between other species. Due to the nature and function of the animal breeding facility, which serves research and educational purposes, the strains studied, including MRSA, could probably be transmitted from an animal to a human (likely by support staff) or vice versa.

## 5. Conclusions

The results of our studies confirmed that farm animals, such as sheep, are still an underestimated reservoir of *S. aureus* strains, including MRSA, VRSA, and MDR strains. Although all the studied individuals were healthy animals, numerous isolates of *S. aureus* were identified in both breeds and we isolated several different phenotypically and genetically distinct strains from the same individual in many cases. Our research also showed that the oral cavity is the most heavily colonized site by *S. aureus* in sheep, regardless of the breed, which is certainly worth considering when conducting monitoring studies in this host species. We confirmed that the Uhruska is more susceptible to *S. aureus* colonization. Moreover, strains isolated from sheep of this breed are characterized by a significantly higher rate of MDR and statistically significantly higher levels of phenotypic resistance to phenicols, aminoglycosides, and macrolides as well as a higher prevalence of certain resistance genes, such as *erm*B and *tet*K. This may indicate that a more primitive breed, such as Świniarka, is more resistant to both colonization and infection, which in turn is associated with less frequent antibiotic use in these animals and lower levels of resistance in the colonizing strains. Interestingly, the presence of MRSA strains was detected at low levels in both breeds. However, we identified three new sequence types out of five strains, which may indicate the emergence of potentially new strains that could pose a threat to public health in the future.

## Figures and Tables

**Table 1:** Occurrence of *S. aureus* by sheep breed and collection site.

Sheep Breed	Source of Isolation				
	Skin	Ear	Rectum	Mouth **	Milk
Uhruska (*n* = 66)	6	4	7	40	9
Świniarka (*n* = 37)	4	3	8	21	1
Total *n* = 103/% *	10/9.7	7/6.8	15/14.6	61/59.2	10/9.7

* percentage of strains in relation to the total number of isolates. ** isolates from ewes and lambs.

**Table 2:** Occurrence of *S. aureus* by sheep breed and collection time.

Sheep Breed	Periods of Collection *			
	I	II	III	IV
Uhruska (*n* = 52)	8	14	18	12
Świniarka (*n* = 36)		15	17	4
Total *n* = 88/%	8/9.1	29/32.9	35/39.8	16/18.2

* I, II, III, IV—time of sample collection: I—before pregnancy, II—at the beginning of pregnancy, III—at the end of pregnancy, IV—after delivery/during lactation.

**Table 3:** Phenotypic resistance of *S. aureus* strains by sheep breed.

Antimicrobials	Breed		Total * n = 103/%
	Uhruska *n* = 66/%	Świniarka *n* = 37/%	
Cefoxitin	2/3	3/8.1	5/4.9
Chloramphenicol **	7/10.6		7/6.8
Ciprofloxacin	5/7.6	2/5.4	7/6.8
Clindamycin	30/45.5	17/45.9	47/45.6
Erythromycin **	22/33.3	19/51.4	41/39.8
Gentamicin **	20/30.3	5/13.5	25/24.3
Linezolid	5/7.6	2/5.4	7/6.8
Rifampicin	1/1.5		1/1
Nitrofurantoin	2/3		2/1.9
Penicillin	23/34.8	9/24.3	32/31.1
Quinupristin /dalfopristin	5/7.6		5/4.9
Sulfamethoxazole/ trimethoprim	1/1.5		1/1
Tetracycline	31/47	17/45.9	48/46.6

* Phenotypic resistance also includes strains isolated from lambs and from milk. ** statistically significant difference (*p* < 0.05) in resistance to antibiotics between *S. aureus* isolates from the Uhruska and Świniarka sheep breeds.

**Table 4:** Occurrence of resistance genes in *S. aureus* strains by sheep breed.

Resistance Genes	Breed		Total * n = 103/%
	Uhruska *n* = 66/%	Świniarka *n* = 37/%	
*mecA*	2/3		2/1.9
*mecC*	3/4.5	2/5.4	5/4.9
*msrA*	3/4.5		3/2.9
*cat (pC194)*	2/3	1/2.7	3/2.9
tetK **	18/48.6	3/4.5	21/20.4
*tetM*	2/3		2/1.9
*tetL*	15/40.5	13/19.7	28/27.2
*blaZ*	8/21.6	4/10.8	12/11.7
*acc(6′)-Ie-*	4/10.8	3/4.5	7/6.8
ermB **	6/16.2		6/5.8

* Occurrence of resistance genes, including strains isolated from the lambs and milk. ** statistically significant difference (*p* < 0.05) in the occurrence of resistance genes between *S. aureus* isolated from the Uhruska and Świniarka sheep breeds.

**Table 5:** Occurrence of virulence genes in *S. aureus* strains by sheep breed.

Virulence Genes	Breed		Total * n = 103/%
	Uhruska *n* = 66/%	Świniarka *n* = 37/%	
*seA*	2/3		2/1.9
*seB*	28/42.4	18/48.6	46/44.7
*seC*	18/27.3	15/40.5	33/32
*seD*		1/2.7	1/1
*seE*		1/2.7	1/1
*tst*		1/2.7	1/1
*pvL*	2/3		2/1.9
*LukE-LukD*	21/31.8	10/27	31/30

* Occurrence of virulence genes, including strains isolated from the lambs and milk.

**Table 6:** Characteristics of methicillin-resistant *S. aureus* strains isolated from the Świniarka breeds (Ś) and Uhruska breeds (UHR).

No. of Isolate/Isolation Period/ADSSR Profile	Source of Isolation	DDT Results	Minimum Inhibitory Concentrations (µg/mL) Results	Resistance and Virulence Genes	Sequence Types (ST)
55 PON */3/R	Mouth	R:E15, P10, SXT25, TE30, C30 I: DA2	R:VA(32), PEN(64), TE(128), C(32), E(8) I: DA(2)	*mecA*, *mecC*, *cat(pC194)*, *tetK*, *seB*, *seC*	9313
78 PON/4/Y lamb	Mouth	R: FOX30, P10, TE30 I: CN10, DA2	R: VA (32), PEN(64), TE(128), OX(8) I: DA(2), CN(8)	*mecA*, *mecC*, *tetM*, *tetK*, *aph(3)-IIIa*, *tetL*, *msrA*, *seB*, *seC*, *Luk E-D*	1660
92 Ś/4/b	Mouth	R: P10 I: CN10, DA2, TE30	R: PEN(128) I: DA(1), VA(8), CN(8), TE(8)	*mecC*, *blaZ*, *acc(6′)Ie*, *seB*, *seC*, *Luk E-D*	9314
93 Ś/4/d	Mouth	R: P10 I: E15, CN10, DA2, TE30	R: VA(64), PEN(128) I: DA(2), CN(8), TE(8), E(4)	*mecC*, *blaZ*, *acc(6′)Ie*, *seB*, *seC*, *Luk E-D*	8420
103 PON/4/J	Milk	R: FOX30, P10 I: CN10, TE30, C30	R: CN(16), PEN(128), C(32), OX(8) I: VA (8)	*mecC*, *tetK*, *aph(3)IIIa*, *tetL*, *acc(6′)-Ie*, *seB*, *seC*	9315

* UHR = PON (abbreviation UHR is the same as the abbreviation PON).

**Table 7:** ADSRRS-fingerprinting profiles by sheep breed and number of animals.

	Profiles with Three or More Strains														Profiles with Two Strains					Profiles with Single Strains
Profile	A	D	E	H	J	K	P	R	S	U	Y	b	c	d	B	M	N	T	X	
Uhruska breed	V (2), VI, X, XVI, XX (2), XXIV, XX,	VII, XX, XXI, XXII, XXIII	II, VIII, XII, XXIV, XXV,	III, IV, XIV	XIX, XIII, XXV	I, IV, XIII, XX, XXIII, XXIV,	II (2), XI	XXIV	VI (3), XIII, XV	IV	IV (2), V, VII, XV, XVII	VI, XVII	III, VII, VIII	IV, IX, XVI, XVII, XXIV,				IV, XVIII	IV, VIII	C-XII, I-VII, Z- VII, a-IX, f-IV, g-XI, h-XIX
Świniarka breed	VII, IX(2), XI, XII, XVI		XXV			I, VII, VIII, IX, XXI, XXV		XV (2)	XXIV (2)	XIX, XXII		II, V		II, XIV,	IV, V	I, IV	I, IV			F-XXI G-XXIV L-XIV O-XVI W-VI, e-VIII

## Data Availability

The original contributions presented in this study are included in the article/Supplementary Material. Further inquiries can be directed to the corresponding author.

## Supplementary Materials

The following are available online at https://www.mdpi.com/article/10.3390/ani16030400/s1, Figure S1: Dendrogram of similarity of *S. aureus* strains tested (ADSRRS-fingerprinting method; Table S1: Occurence of *S. aureus* strains by animal and collection time and site.
