# Supplementary material for: Perinatal Occurrence and Epidemiological Significance of Staphylococcus aureus in Local Sheep Breeds
Source: Animals (Basel). 2026 Jan 27;16(3):400. doi: 10.3390/ani16030400 (PMC12897316; doi:10.3390/ani16030400)
Supplement: Supplementary file 1 [file animals-16-00400-s001.zip › animals-4087616-supplementary.pdf]

Table S1. Occurence of *S. aureus* strains by animal and collection time and site

Sheep breed	No of animal	Period of collection			
		I	II	III	IV
Uhruska (n=24)	1			M*	
	2		M**		S
	3		M	M	E
	4		R		M
	5	M	M		
	6	M	M		
	7		M		
	8	M	S		
	9				R,M
	10	M			
	11				E
	12		S	E,M	
	14		M	M	M
	15			M,R	M
	16		S	R,M	
	17			S	M
	18			M	
	20	M	S,R	M	
	21	E			
	22		S	M	
	23	M		M,R	M
	24		M	R	
	25	M		M	M
	28			M	M
Świniarka (n=18)	1			M,R,E	
	2		R,S		
	4		E	R,M	
	5		S,M		
	6		M		
	7		S	M	
	8		M	R	
	9		E,S	M	
	11		M		
	12			M	
	14		R	M	
	15			M	M
	16		M	R	
	19			M	
	21		M	R,M	M
	22		M		
	24			M	
	25		M	M,R	

*Site of isolation: M: mouth, E: ear canal, S: skin, R: rectum
**Isolates marked red had the same ADSSRS profile in the same animal, but were isolated from different sites or differed slightly in resistance and/or virulence profiles

***I, II, III, IV- time of sample collection: I- before pregnancy, II- at the beginning of pregnancy, III- at the end of pregnancy, IV- after delivery/during lactation

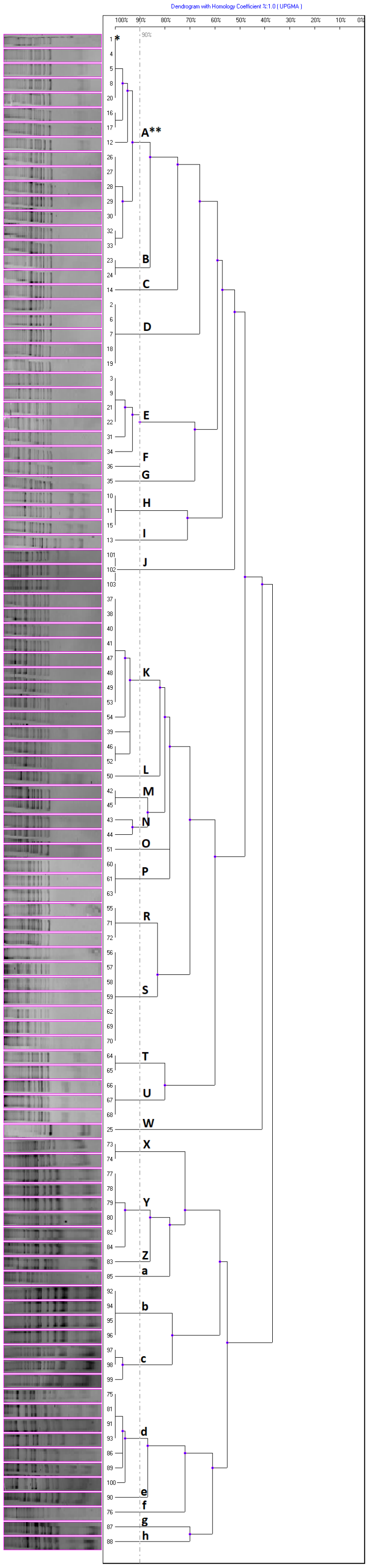

Figure S1.Dendrogram of similarity of *S. aureus* strains tested (ADSRRS-fingerprinting method)

*Strain numbers are in the order of their isolation.

**ADSSR fingerprinting profile designations
